# Psycho-Therapeutics, or Treatment by Sleep and Suggestion

**Published:** 1889-12

**Authors:** 


*Psycho-therapeutics, or Treatment by Sleep and Suggestion.* By C. LLOYD TUCKEY, M.D. Pp. 80. London: BAILLIÈRE & Co. 1889.

As we have no personal experience of the therapeutic virtues of hypnotism, we do not dare to criticise the work before us. In its favour we can say that it is clearly written in a calm, almost judicial, spirit, and that it can scarcely fail to carry some degree of conviction to the mind of the reader. The author has studied the subject at first hand under the best masters on the Continent, and has had some practical successes from the method in London.

Should this new therapeutic agent prove valuable and come into repute, there must be men even now living who will feel uncomfortable qualms of conscience at the manner in which they hounded to disgrace and death a certain physician who dared openly to practise it. Religion has not been the only persecutor of Science.

